# *Vaccinium bracteatum* Thunb Extract Inhibits HSV-1 Infection by Regulating ER Stress and Apoptosis

**DOI:** 10.3390/antiox11091773

**Published:** 2022-09-08

**Authors:** Buyun Kim, Eun-Bin Kwon, Hye Jin Yang, Wei Li, Youn-Hwan Hwang, Young Soo Kim, Malk Eun Pak, Younghoon Go, Jang-Gi Choi

**Affiliations:** Korean Medicine (KM) Application Center, Korea Institute of Oriental Medicine (KIOM), Dong-gu, Daegu 701-300, Korea

**Keywords:** herpes simplex virus type 1, antiviral effect, *Vaccinium bracteatum* Thunb, reactive oxygen species, ER stress, apoptosis

## Abstract

Herpes simplex Type 1 (HSV-1) is a neurotropic virus that infects the peripheral and central nervous system. Usually, after primary infection in epithelial cells, HSV-1 migrates retrograde to the peripheral nervous system (PNS), where it establishes a latent infection. HSV-1 can remain latent in the nervous system, and its reactivation in the brain can rarely cause acute HSV-1 encephalitis, often a life-threatening condition, or asymptomatic reactivations that could lead to neuronal damage and ultimately neurodegenerative disorders. Acyclovir and related nucleoside analogs have been used as therapeutic agents for HSV-1 infection, but resistance to the drug can arise, and the protective effect of HSV-1 on brain cells is limited. Therefore, there is an urgent need for research into safe and effective new antiviral agents that can protect brain cells from the damage that is caused by HSV-1 infection. *Vaccinium bracteatum* Thunb. (VBT) is widely distributed in Korea and China, and has pharmacological actions such as anti-inflammatory, antioxidant, and antidiabetic activity. Studies on the antiviral effect of VBT on HSV-1 infection have not been reported so far. Therefore, we sought to determine the HSV-1 antiviral effect and molecular mechanism of VBT at the cellular level. We confirmed that VBT repressed the VP16 and IE genes in both Vero and SK-N-SH cells. We also found that the generation of HSV-1 virions was inhibited by VBT treatment. VBT inhibited the activities of the HSV-1-induced endoplasmic reticulum (ER) stressors PERK, ATF4, and CHOP. We confirmed that VBT inhibited the activity of apoptosis factors by regulating the expression of death receptor (DR) after HSV-1 infection. As HSV-1 is closely associated with brain diseases, the study of the antiviral drug effects and mechanism of VBT is meaningful. Further studies using animal models of infection will also be performed to determine the potential of VBT as an antiviral agent.

## 1. Introduction

Herpes simplex virus Type 1 (HSV-1), a member of the Herpesviridae family, is one of the most prevalent human pathogens, and can produce a range of diseases, including neuropathy [1]. HSV-1 causes a primary infection of the ocular or oral surfaces, followed by lifelong latent infection of neurons of the central nervous system and sensory neurons of the trigeminal ganglion (TG). Therefore, HSV-1 is a common human neurogenic virus that accounts for more than 90% of herpes simplex encephalitis (HSE) cases [2]. HSE causes severe impairment of nerve function, usually associated with the frontal and temporal lobes, leading to clinical features such as personality changes, cognitive impairment, aphasia, seizures, and weakness in focus [3,4]. In addition, persistent viral recurrence of HSV-1 has been reported to cause local nerve damage and cause neurodegeneration and Alzheimer’s disease (AD) [5,6,7].

The HSV-1 gene is expressed in a tightly regulated transient cascade involving the sequential expression of immediate early (IE), early (E), and late (L) during lytic infection. IE proteins include ICP0, ICP4, ICP5, ICP27, and ICP 34.5 and are involved in viral early gene expression and host cell protein regulation [8,9]. Among them, ICP27 is a multifunctional protein that is essential for viral replication by being involved in cell cycle regulation and activation of signaling pathways and plays a pivotal role in the transition from early gene expression to late gene expression [8]. In addition, ICP27 is well known to play an important role in the induction of apoptosis in HSV-1-infected cells by promoting the mitochondrial translocation of Bax [9].

Cells that are infected with HSV-1 show a cytopathic effect (CPE), and are generally observed to be closely agglomerated immediately after infection [10]. CPE damages the neurons and other brain cells, leading to apoptosis, either intrinsically or via mediators of the host immune response [11]. Interactions between HSV-1 and host cells result in the triggering of the apoptotic cell death program [12,13,14]. Apoptosis is known to be induced by endoplasmic reticulum (ER) stress [15] and the generation of reactive oxygen species (ROS) [16,17]. The influx of peripheral lymphocytes during acute HSV-1 infection of the brain helps clear the virus. This influx of lymphocytes is known to recruit astrocytes and microglia, which are resident cells of the central nervous system, and secrete specific cytokines and chemokines to respond to viral infection [18]. Specific pattern recognition receptors in microglia and astrocytes recognize invariant HSV-1 structures such as proteins or nucleic acids, known as pathogen-associated molecular patterns (PAMPs) [19]. PAMP recognition also induces the expression of nitric oxide synthetase and reactive oxygen species (ROS) in glial cells, leading to massive inflammation [16,20]. The generation of ROS in response to infection is closely related to ER stress, and previous studies have demonstrated the importance of ER stress and the unfolded protein response (UPR) during viral infection. Since a large number of viral proteins are synthesized in virus-infected host cells, misfolded or completely unfolded proteins are produced [15,21]. The production and processing of viral proteins in the ER in HSV-1-infected human and mouse cells induces the oligomerization and activation of PERK, due to increased autophosphorylation [22,23]. Upon HSV-1 infection, eIF2α/ATF4 signaling is activated at the final stage of HSV-1 replication, which may indicate the completion of virion assembly and escape [24]. Activated ATF4 activates C/EBP homologous protein (CHOP), a transcription factor which controls genes that are involved in the apoptosis pathway [25]. Therefore, HSV-1 infection is associated with neurological diseases, and therapies that are aimed at the treatment and prevention of neurological diseases may include the use of antiviral agents and the targeting of HSV-1 activating intracellular pathways.

Acyclovir and related nucleoside analogs have been used as therapeutic agents for HSV-1 infection, but resistance to the drug can arise, and the protective effect of HSV-1 on brain cells is limited [26]. For example, ACV-refractory HSV keratitis has been reported as a leading cause of corneal morbidity and blindness in humans in industrialized countries [27]. In addition, the prevalence of ACV resistance is particularly high in immunocompromised patients that are receiving long-term prophylactic treatment with ACV [28]. This is because resistance to ACV is mostly acquired through mutation of the HSV thymidine kinase (TK) gene. Therefore, cross-resistance to other viral TK-dependent nucleoside analogs such as penciclovir and famciclovir is not uncommon [26,29]. Therefore, there is a need for research into safe and effective new antiviral agents that can protect brain cells from damage that is caused by HSV-1 infection.

*Vaccinium bracteatum* Thunb. (VBT) is an evergreen shrub or small tree belonging to the family Rhododendron, and is found in Korea and China [30]. VBT is used in medicine and as a natural dye for protein, hair, and starch. VBT has been reported to have antioxidant [31], anti-inflammatory [32], antibacterial [33], and antidiabetic activity [34]. VBT contains ingredients such as orientin, vitexin, lysine, apigenin, and kaempferol, which are known to have antioxidant activity [31]. However, the antiviral effects of VBT on HSV-1, and the underlying cellular mechanisms, have not yet been investigated. In this study we investigated the antiviral effects of VBT and the mechanisms underlying its protective role against neuronal cell death following HSV-1 infection. We found that VBT had antiviral activity and was effective against HSV-1. We suggest that VBT may protect neurons from HSV-1 infection by inhibiting the induction of ER stress and the apoptosis of neurons following HSV-1 infection.

## 2. Materials and Methods

### 2.1. Materials

A Vero African green monkey kidney cell line (CCL-81) and SK-N-SH neuroblastoma cells (HTB-11) were purchased from the American Type Culture Collection (ATCC; Manassas, VA, USA). Dulbecco’s modified Eagle medium (DMEM), fetal bovine serum (FBS), and the antibiotic-antimycotic mixture were obtained from Gibco BRL (Grand Island, NY, USA). The HSV green fluorescent protein (HSV GFP) strain was purchased from Imanis Life Sciences (Rochester, MN, USA), and the KBPV-VR-733 (HSV-1 strain) was obtained from the Korea Pathogenic Virus Bank. HSV-GFP virus, only the part confirming antiviral efficacy was used, and additionally, the antiviral efficacy and the antiviral mechanism of action were tested using the HSV-1 strain KBPVVR-733. In February 2001, dried stems of *Vaccinium bracteatum* Thumb were collected from Jeju Island, Korea, and the voucher specimen (001-096) was deposited with the Korean Plant Extracts Bank in Cheongju, Korea. The dried VBT leaves were extracted three times with MeOH (99.9%; HPLC grade) under reflux to obtain an extract in a yield of 19.9%. Finally, the total yields of methanol extracts were obtained by freeze-drying, and VBT sample was dissolved with a final concentration of 100 mg/mL in DMSO for subsequent bioassays. All the reference standards that were used for UHPLC-UV-HRMS analysis, such as caffeic acid, p-coumaric acid, epicatechin, orientin, and isoorientin, were purchased from Sigma-Aldrich Co. (St. Louis, MO, USA). The purity of all the reference standards was above 97%. Water, acetonitrile, and formic acid, used as the mobile phase, were MS-grade products that were purchased from Thermo Fisher Scientific (Pittsburgh, PA, USA). The antibodies ICP0 (1:5000; mouse, monoclonal, cat. no.H1A027), ICP4 (1:5000; mouse, monoclonal, cat. no.H1A021), ICP5 (1:5000; mouse, monoclonal, cat. no. HA018), and ICP27 (1:5000; mouse, monoclonal, cat. no. p1113) were purchased from Virusys Corporation (Virusys, Sykesville, MD, USA). ICP34.5 antibody (1:1000; rabbit, polyclonal, cat. no. p1113) was obtained from Abnova (Abnova, Taipei City, Taiwan). Bip (1:1000; rabbit, polyclonal, cat. no. 3183), PERK (1:1000; rabbit, polyclonal, cat. no. 3140), ATF4 (1:1000; rabbit, polyclonal, cat. no. 11815), CHOP (1:1000; mouse, monoclonal, cat. no. 2895), cleaved caspase-9 (1:1000; rabbit, polyclonal, cat. no. 20750), cleaved caspase-3 (1:1000; rabbit, polyclonal, cat. no. 9661), cleaved PARP (1:1000; rabbit, polyclonal, cat. no. 9541), cytochrome C (1:1000; rabbit, polyclonal, cat. no. 4280), BAX (1:1000; rabbit, polyclonal, cat. no. 2772), Bcl-2 (1:1000; rabbit, polyclonal, cat. no. 3498), and anti-rabbit IgG (1:5000; rabbit, polyclonal, cat. no. 14708) were obtained from Cell Signaling Technology Inc. (Danvers, MA, USA). β-actin (1:1000; mouse, monoclonal, cat. no. sc-81178) and goat anti-mouse IgG (1:5000; mouse, monoclonal, cat. no. sc-2355) were purchased from Santa Cruz Biotechnology (Santa Cruz, CA, USA). Anti-HSV1 + HSV2 VP16 (1:10,000; mouse, monoclonal, cat. no. ab-110226), Anti DR5 (1:5000; rabbit, polyclonal, cat. no. ab8416) and Anti DR4 (1:5000; rabbit, polyclonal, cat. no. ab216662) were obtained from Abcam (Cambridge, MA, USA). p-PERK(Thr982) (1:1000; rabbit, polyclonal, cat. no. PA5-102853) was purchased from Invitrogen (Groningen, The Netherlands).

### 2.2. Cell Cultures and Viruses

Vero cells were propagated in Dulbecco’s Modified Eagle Medium (DMEM) that was supplemented with 10% fetal bovine serum (FBS) and 1% penicillin/streptomycin (100 U/mL). SK-N-SH were cultured in minimum essential medium that was supplemented with nutrients and essential conditions, according to the manufacturer’s instructions (GIBCO, Invitrogen Corporation; Grand Island, NY, USA). The cells were maintained at 37 °C in a CO_2_ incubator in a saturated humidity atmosphere containing 95% air and 5% CO_2_. The HSV-1 strains (KBPV-VR-733) and HSV GFP strains were stored at −80 °C, and were slowly thawed on ice before use.

### 2.3. In Vitro Cytotoxicity Assays

For cytotoxicity experiments by HSV-1, a Cell Counting Kit-8 (CCK-8, Dojindo, Kumamoto, Japan) was used. Vero and SK-N-SH cells were seeded in 96-well plates at a density of 5 × 10^4^ cells per well. The next day, the cells were infected with HSV-1 (MOI = 0.1) for 2 h, then replaced with fresh medium, and VBT was incubated at various concentrations (min. 1.5 μg/mL to max. 200 μg/mL) for 48 h. Then, 10 μL of CCK-8 reagent was treated in each well, and the plate was wrapped in silver foil and incubated at 37 °C for 3 h. The absorbance was measured at 450 nm using a GloMax^®^ Explorer Multimode Microplate Reader (Promega). Cell viability was calculated as the percentage of viable cells compared to the mock-treated control cells.

### 2.4. Analysis of HSV-1 GFP Expression

To check the VBT inhibitory effect according to HSV GFP infection, Vero and SK-N-SH cells were inoculated into a 24-well plate at 1 × 10^5^ cells/well. The next day, the cells were treated with HSV-1 GFP (MOI = 2) and then infected at 37 °C for 2 h. The infected medium was removed by washing 3 times with PBS. Afterwards, the fresh medium was treated with 50 and 100 µg/mL of VBT and 10 µg/mL of acyclovir (ACV) as positive controls, followed by incubation at 37 °C for 48 h. Green fluorescence expression after HSV-GFP infection was imaged using a fluorescence microscope (Nikon ECLIPSE Ti-U, Nikon Co., Tokyo, Japan). The GFP expression rates were also measured using a CytoFLEX flow cytometer (Beckman Coulter Inc., Pasadena, CA, USA) and FlowJo software. The cells were collected in 1.5 mL tubes and then washed with PBS. They were then fixed in suspension containing 4% paraformaldehyde (PFA). Again, the cells were washed with PBS and analyzed for green fluorescence expression.

### 2.5. Plaque Reduction Assay

The antiviral activity of VBT was measured using a plaque reduction assay upon HSV-1 infection. The Vero cells were seeded at 5 × 10^5^ cells/well in 6-well plates. The cells were infected with HSV-1 at MOI = 0.1 concentration at 37 °C for 2 h, and then washed with PBS to remove the inoculum. VBT (50 μg/mL and 100 μg/mL) and ACV (10 μg/mL) were incubated on a 1.5% agarose gel (diluted in 2xDMEM) for 4 days. After fixing for 20 min at room temperature with 4% paraformaldehyde (PFA), the agar was removed after staining with 1% crystal violet for 30 min. The resulting plaques corresponding to each group were imaged and the number of plaques was quantified.

### 2.6. Transmission Electron Microscopy

The HSV-1 (MOI = 0.1)-infected Vero cells were treated with 100 μg/mL VBT for 48 h. The cells were collected when 70–80% of the model group showed obvious cytopathic effects (CPE). Then, they were fixed at least for 2 h with 2% glutaraldehyde in 0.05 M sodium carcodylate buffer. After the reaction, the cells were washed three times with 0.05 M sodium carcodylate buffer and then post-fixed with 1% osmium tetroxide in 0.05 M sodium carcodylate buffer for 1 h. All fixation occurred at 4 °C. The cells were En-bloc stained with 0.5% uranyl acetate at 4 °C overnight. After incubation, the cells were gradually dehydrated using 40%, 50%, 60%, 80%, 90%, and 100% ethanol at 25 °C for 10 min, respectively. The cells were then treated with 100% propylene oxide at 25 °C for 15 min, which lead to infiltration and polymerization. Slices were obtained using an ultramicrotome following 2% uranyl acetate staining. Electron micrographs were obtained using an SU5000 SEM (Hitachi High-Tech, H-7650, Tokyo, Japan).

### 2.7. Immunofluorescence Staining

The expression of ICP0 and ICP27 in SK-N-SH cells was determined using immunofluorescence assays. SK-N-SH cells were seeded on glass coverslips in 4-well plates. Then, HSV-1 (MOI = 0.1) was infected for 2 h, and VBT 100 μg/mL and ACV 10 μg/mL were treated in fresh medium and cultured for 48 h. After that, PBS was washed 3 times and fixed with 4% PFA. Then, after treatment with 0.1% Triton X-100 for 5 min, it was washed 3 times with PBS. Then, primary monoclonal anti-ICP27 or ICP0 antibody (dilution, 1:500; Virusys, Sykesville, MD, USA) was reacted for 1 h at RT. After removing the primary antibody and washing three times with PBS, the cells were reacted with a secondary antibody (Alexa Flour 488, Thermo Fisher Scientific, Waltham, MA, USA) for 30 min. Then, for nuclear staining, it was reacted with Hoechst 33342 (ImmunoChemistry, Bloomington, MN, USA) at 37 °C. for 10 min. Slides were fixed using ProLong^®^ Gold anti-discoloration reagent (Molecular Probes^®^ from Life Technologies, Carlsbad, CA, USA) and then fluorescence pictures were taken using a fluorescence microscope (Nikon ECLIPSE Ti-U, Nikon Co., Tokyo, Japan).

### 2.8. Determination of Intracellular Levels of ROS

The levels of ROS that were induced by HSV-1 infection were measured using 2,7-dichlorofluorescein diacetate assays (DCFH-DA). To perform the assay, 5 × 10^5^ cells were plated into 6-well culture wells. Two hours after infection with HSV-1 (MOI = 0.01), the cells were washed with PBS and fresh medium was added. After incubation for 24 h at concentrations of VBT extract of 50 μg/mL and 100 μg/mL, 10 μM DCFH-DA was added to the cells, and the suspension was incubated for an additional 30 min. The ROS value was photographed with a fluorescence microscope, and then cells were collected and the fluorescence was measured at an excitation wavelength (Ex) of 485 nm and an emission wavelength (Em) of 530 nm, using a CytoFLEX flow cytometer (Beckman Coulter Inc., Pasadena, CA, USA).

### 2.9. Live/Dead Assays

Apoptosis was measured using the LIVE/DEAD assay, a two-color fluorescence assay that determines the number of live and dead cells. Briefly, 5 × 10^5^ SK-N-SH cells were infected with HSV-1 for 2 h and then incubated with VBT drugs at concentrations of 50 μg/mL and 100 μg/mL at 37 °C for 48 h. The cells were stained with LIVE/DEAD reagent (5 μM ethidium homodimer and 5 μM calcein-AM) and incubated at 37 °C for 30 min. The cells were imaged using a fluorescence microscope at ×200 magnification (Nikon Corporation, Tokyo, Japan).

### 2.10. RNA Isolation and Quantitative Real-Time PCR Analysis

To isolate RNA, the cells were lysed in Trizol (Invitrogen) and phase separated by the addition of chloroform and centrifugation at 12,000× *g* for 15 min at 4 °C. The aqueous phase was isolated, and RNA was precipitated with isopropanol and pelleted by centrifugation for 10 min at 10,000× *g* at 4 °C. cDNA was synthesized by Maxima First Strand cDNA Synthesis Kit for RT-qPCR ((Thermo Fisher Scientific, Waltham, MA, USA). The expression of relative genes with PowerUp™ SYBR^®^ Green master mix was measured by using a Real-Time PCR System 7500 (Applied Biosystems, Foster City, CA, USA). The primer sets that were used for each gene were as follows: GAPDH, 5′-CAAGAAGGTGGTGAAGCAGGC-3′ and 5′-CATACCAGGAAATGAGCTTGAC-3′ (Gene bank accession number: NM_001 357943.2); ICP0, 5′-CGTCTTGTTCACGTAAGGCG-3′ and 5′-GAGGAAGTGTGCCAGGAAGA-3′ (Gene bank accession number:MN401201.1); ICP27, 5′-CCCTTTCTGCAGTGCTACCT-3′, and 5′-CCTTAATGTCCGACAGGCGT-3′ (Gene bank accession number: MN_401207.1); GB, 5′-GGACATCAAGGCGGAGAACA-3′ and 5′-TTCTCCTTG AAGACCGC -3′ (Gene bank accession number: MN401208.1). GAPDH was used as an internal control with conditions of 95 °C for 3 min, followed by 40 cycles of 95 °C for 10 s, and 55 °C for 30 s. The expression difference was calculated on the basis of 2^−ΔΔCt^ values.

### 2.11. Western Blot Assay

For protein extraction, RIPA buffer (Thermo Scientific, Waltham, MA, USA) that was supplemented with proteinase inhibitor cocktail and phosphatase inhibitor cocktail was used. The protein concentration was determined by BCA assay kit (Thermo Scientific). Equal amounts of protein were loaded, subjected to SDS-PAGE, and transferred to PVDF membranes (Millipore, Boston, MA, USA). After blocking with 3% BSA for 1 h at room temperature, it was washed 3 times with PBS for 10 min. The PVDF membranes were incubated with primary antibody overnight at 4 °C. The antibodies that were used in our study are indicated in Section 2.1 material. The membranes were then incubated with secondary antibodies and visualized with ECL (Thermo Scientific, Rockford, IL, USA). Each band intensity was calculated using ImageJ.

### 2.12. Ultra-High Performance Liquid Chromatography Coupled with High-Resolution Orbitrap Mass Spectrometry

To identify the phytochemicals in *Vaccinium bracteatum*, analysis was performed using a Thermo Dionex UltiMate 3000 system, coupled with a Q-Exactive orbitrap mass spectrometer (UHPLC-UV-HRMS, Thermo Fisher Scientific, San Jose, CA, USA). The analysis method that was used in this study was previously reported [26,27]. Briefly, chromatographic separation was performed using a Waters Acquity BEH C18 column (100 × 2.1 mm, 1.7 µm) with a VanGuard XBridge^®^ BEH C18 pre-column (2.1 × 5 mm, 1.7 µm), and a mobile phase consisting of 0.1% formic acid (*v*/*v*) in water (A) and acetonitrile (B). The flow rate was maintained at 0.3 mL/min. MS/MS analysis was conducted using a Q-Exactive quadrupole-Orbitrap mass spectrometer that was equipped with a heated electrospray ionization interface. All data acquisition and analysis were performed using Xcalibur v.4.2 and Tracefinder v.4.0 software (Thermo Fisher Scientific) [See Supplementary Materials].

### 2.13. Statistical Analysis

All experimental data are presented as the mean (±SEM) and standard deviation of at least 3 independent experiments (n). Statistical analysis was performed using GraphPad Prism 6.0 software. One-way analysis of variance (ANOVA) and Tukey multiple comparisons were performed to test for significant differences between the means. The dr4ifferences were considered statistically significant at *p*-values < 0.05.

## 3. Results

### 3.1. VBT Inhibits Cytotoxicity and Viral Activity upon HSV-1 Infection in Vero Cells

**Figure 1:**
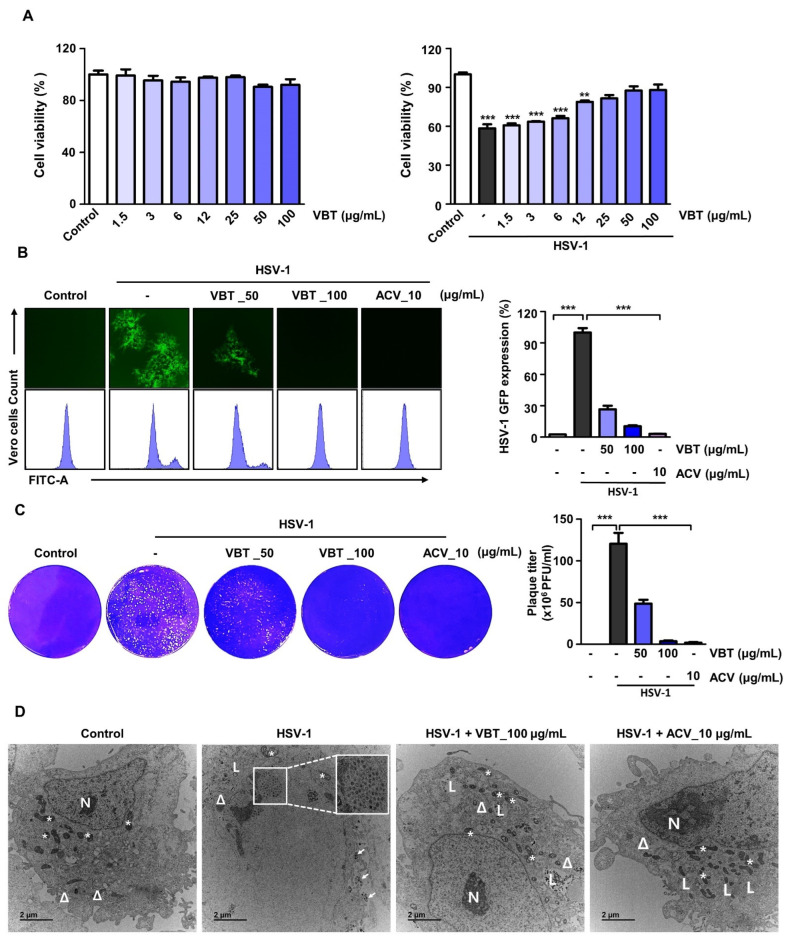
Anti-HSV-1 activity of VBT in Vero cells. (**A**) Vero cells were treated with VBT at the indicated concentrations and incubated for 48 h (**Left**). VERO cells were infected with HSV-1 (MOI = 0.1) for 2 h, then replaced with fresh medium, treated with VBT at the indicated concentration, and incubated for 48 h (**Right**). Cell viability was measured using CCK-8 assays. (**B**) Vero cells were infected with HSV-GFP (MOI = 2) for 2 h, then the medium was replaced with fresh medium, and treated with VBT (50 µg/mL and 100 µg/mL) and positive control ACV (10 µg/mL) for 48 h. HSV-GFP expression levels were analyzed using fluorescence microscopy (**left**) and FACS (**right**). (**C**) VERO cells were infected with HSV-1 (MOI = 0.1) for 2 h, then VBT (50 µg/mL and 100 µg/mL) and ACV (10 µg/mL) were incubated on 1.5% agarose gel for 4 days. The cells were then fixed in 4% PFA and stained with crystal violet dye. Plaque inhibition was calculated by counting plaque numbers and data represent the mean ± SED (n = 3). (**D**) Vero cells were infected with HSV-1 (MOI = 0.1) for 2 h, and then incubated with 100 µg/mL VB or 10 µg/mL ACV for 48 h. The samples were analyzed using TEM. White box frames demarcate the enlarged areas. White arrows indicate HSV-1 viral particles. N; Nuclear, *; Mitochondria, Δ; Endoplasmic reticulum, L; Lysosome, Scale bars, 2 µm. ** *p* < 0.01, *** *p* < 0.001.

We first investigated the antiviral effect of VBT using Vero cells. Vero cells are genetically similar to human cells, so they are easily infected with human viruses. They do not secrete the inflammatory cytokine interferon gamma, so they can maintain high concentrations of viruses, and are optimal cells for virus experiments [35]. First, we indicated that VBT alone treatment did not affect cell viability in Vero cells (Figure 1A, Left). In order to confirm the antiviral effects of VBT following HSV-1 infection in Vero cells, the toxicity was evaluated using CCK-8 assays. We found that VBT restored the reduction in cell viability due to HSV-1 infection at 50 and 100 μg/mL (Figure 1A, Right). Therefore, we decided to perform further experiments on the antiviral effects of VBT on HSV-1 using 50 μg/mL and 100 μg/mL concentrations of VBT. The inhibition of virus replication by VBT was observed in Vero cells that were infected with HSV-1 h HSV-1 green fluorescent protein (GFP). When HSV-1 GFP expression was observed through a fluorescence microscope, it was confirmed that its expression was inhibited at VBT concentrations of 50 μg/mL and 100 μg/mL (Figure 1B, Left). When the HSV-GFP expression was measured using flow cytometry, it was apparent that QA significantly reduced the expression of HSV-GFP (Figure 1B, Right). Plaque experiments were performed to investigate the effect of VBT on the replication of HSV-1 in Vero cells. While plaque formation was observed during HSV-1 infection, it was inhibited by VBT treatment (Figure 1C). Using TEM analysis, we investigated whether VBT inhibited the cytopathic effect. The TEM results showed that VBT promoted the clearance of HSV-1 particles from cells and maintained the structural integrity of the cells (Figure 1D). These results suggest that VBT protects against cytotoxicity and has potent antiviral activity against HSV-1. Next, we checked whether the antiviral effect also occurred in neuronal cells.

### 3.2. VBT Inhibits mRNA and Protein Expression of Genes Involved in HSV-1 DNA Replication and Proliferation in Vero Cells

**Figure 2:**
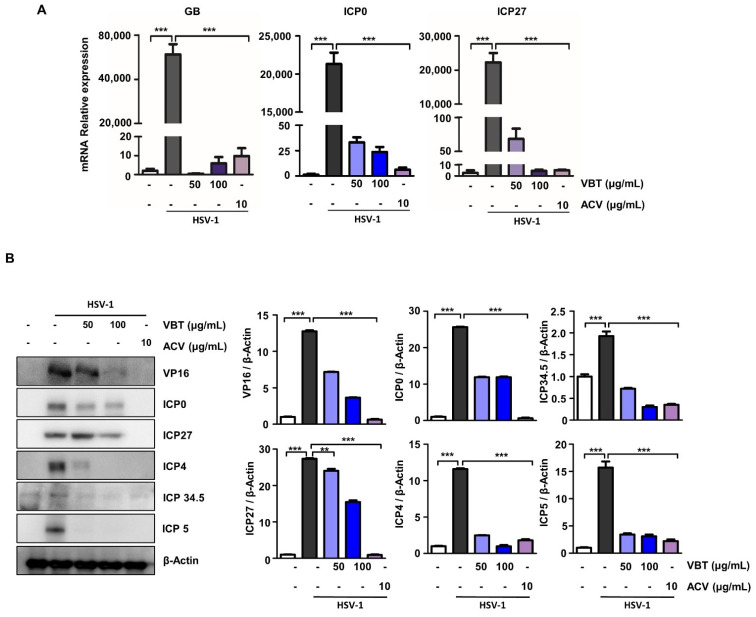
Anti-HSV-1 DNA replication and gene expression of VBT in Vero cells. The cells were infected with HSV-1 (MOI = 0.1) for 2 h and then replaced with fresh medium. Then, they were treated with VBT (50 μg/mL and 100 μg/mL) and ACV (10 μg/mL), and incubated for 48 h. (**A**) Virus-associated factor mRNA levels were identified by real-time PCR. The expression of these proteins was normalized to that of GAPDH as a relative ratio. (**B**) Virus-associated factor proteins were identified by Western blotting. The expression of these proteins was normalized to that of β-Actin as a relative ratio. ** *p* < 0.01, *** *p* < 0.001 vs. HSV-1 treatment cells.

The replication of HSV begins with the introduction of the virus into the cell through fusion of the virus with the cell membrane [36]. HSV-1 gB is a viral envelope glycoprotein that is required for efficient viral entry and is known to bind heparan sulfate proteoglycans on the surface of host cells [37]. One envelope protein, VP16, is a transcriptional activator that binds to cellular transcription factors and enhances the transcription of viral immediate early (IE) genes, thereby enhancing the production of progeny viruses [38]. Therefore, we investigated whether VBT repressed the expression of these genes in HSV-1-infected Vero cells. First, we confirmed that the mRNA expression of GB, ICP0, and ICP27 was significantly reduced by VBT during HSV-1 infection (Figure 2A). In addition, we indicated that the protein expression of VP16, ICP0, ICP27, ICP4, ICP34.5, and ICP5 by HSV-1 infection was also suppressed at the protein level even at the VBT 100 μg/mL concentration (Figure 2B).

We demonstrated that HSV-1 infection releases a number of envelope proteins that are present in virions into infected cells, and that these envelope proteins activate viral replication through various mechanisms, but are inhibited by VBT.

### 3.3. VBT Induces Antiviral Activity by HSV-1 Infection in SK-N-SH Cells

**Figure 3:**
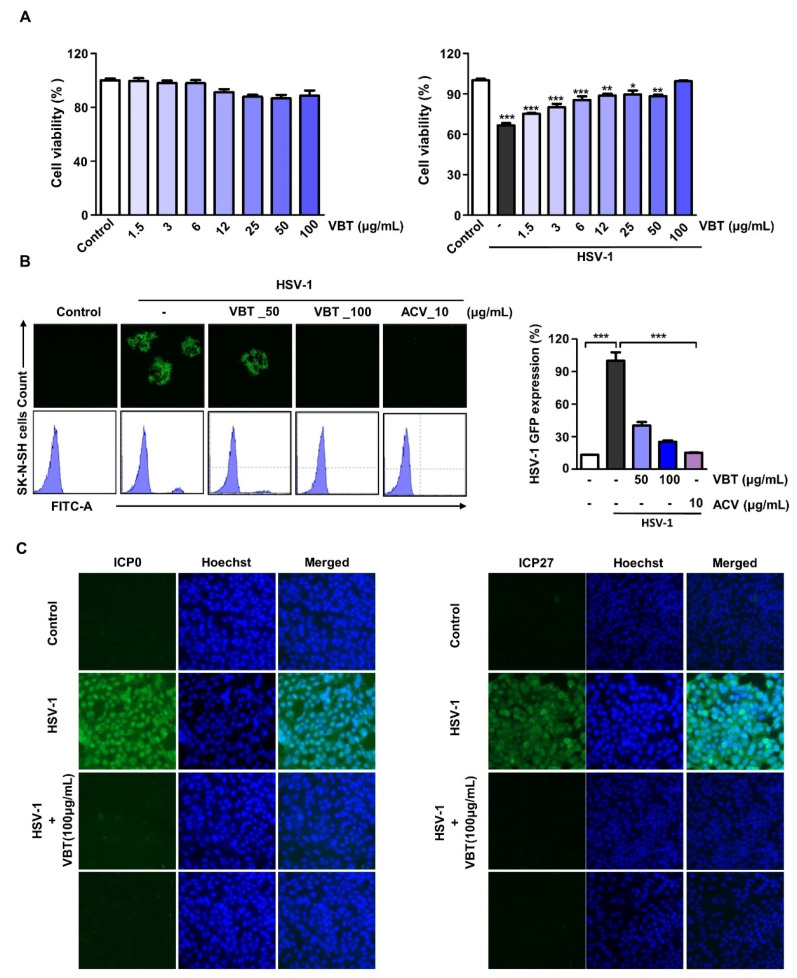
Anti-HSV-1 activity of VBT in SK-N-SH cells. (**A**) SK-N-SH cells were treated with VBT at the indicated concentrations and incubated for 48 h (**Left**). The SK-N-SH cells were infected with HSV-1 (MOI = 0.1) for 2 h (**right**), then replaced with fresh medium, treated with VBT at the indicated concentration, and incubated for 48 h. Cell viability was measured using CCK-8 assays. (**B**) The SK-N-SH cells were infected with HSV-GFP (MOI = 2) for 2 h and then treated with VBT (50 μg/mL and 100 μg/mL) and ACV (10 μg/mL) in fresh medium. Then, after being incubated for 48 h, the HSV-GFP expression levels were analyzed using fluorescence microscopy (**left**) and FACS (**right**). (**C**) ICP0 and ICP27 localization was determined by immunofluorescence assays using a fluorescence microscope. * *p* < 0.5, ** *p* < 0.01, *** *p* < 0.001.

We investigated whether VBT inhibited the cytotoxicity that was caused by HSV-1 infection in the neuroblastoma SK-N-SH cell line, and found that VBT inhibited HSV-1 infection-induced cytotoxicity in a concentration-dependent manner, even in SK-N-SH cells (Figure 3A, Right). In SK-N-SH cells, VBT did not show a toxic effect (Figure 3A, left). After infection with HSV-1 GFP, virus inhibition by VBT in SK-N-SH cells was confirmed using fluorescence images and FACS. VBT decreased the expression of HSV-1 GFP at a concentration of 100 µg/mL, and the expression rate was similar to that of ACV, a positive control (Figure 3B). The HSV-1 immediate early (IE) gene is thought to be important in determining the outcome of infection because it plays a role in cell permissivity, the establishment and reactivation of latency, and viral replication [39]. Therefore, one of the initial requirements for the prevention of neuronal infection is the suppression of viral replication by suppressing the expression of the IE gene [40]. We investigated whether VBT inhibited the expression of ICP0 and ICP27 following HSV-1 infection using the IF method. We found that the proteosynthesis of ICP0 (left) and ICP27 (right) were inhibited by VBT in SK-N-SH cells during HSV-1 infection (Figure 3C).

### 3.4. VBT Inhibits the Expression of Genes Involved in HSV-1 DNA Replication and Proliferation in SK-N-SH Cells

**Figure 4:**
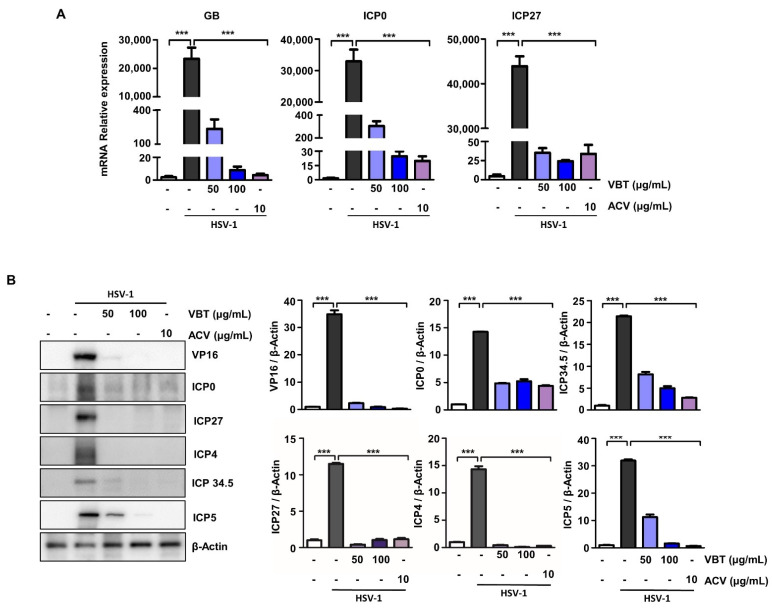
Anti-HSV-1 DNA replication and gene expression of VBT in SK-N-SH cells. The cells were infected with HSV-1 (MOI = 0.1) for 2 h and then replaced with fresh medium. Then, they were treated with VBT (50 μg/mL and 100 μg/mL) and ACV (10 μg/mL), and incubated for 48 h. (**A**) Virus-associated factor mRNA levels were identified by real-time PCR. The expression of these proteins was normalized to that of GAPDH as a relative ratio. (**B**) Virus-associated factor proteins were identified by Western blotting. The expression of these proteins was normalized to that of β-Actin as a relative ratio. *** *p* < 0.001 vs. HSV-1 treatment cells.

During productive infection, it is activated sequentially IE, early and late by the activation of VP16, an envelope protein that forms a complex with host cell factors [41]. To determine the mechanism underlying VBT inhibition of HSV-1 infection, we investigated whether VBT could block HSV-1 viral DNA replication and gene expression. The K-N-SH cells were infected with HSV-1 at an MOI of 0.1 for 2 h, treated with VBT at different concentrations, and then cultured for 48 h. In the SK-N-SH cells, VBT suppressed mRNA production by the GB and IE genes ICP0, and ICP27 following HSV-1 infection (Figure 4A). In SK-N-SH cells, VBT also suppressed protein production by the VP16 and IE genes ICP0, ICP5, ICP 34.5, ICP4, and ICP27 following HSV-1 infection (Figure 4B). These data demonstrated that VBT not only inhibits the replication of HSV-1 viral DNA, but also blocks HSV-1 viral gene expression at the mRNA and protein levels.

### 3.5. VBT Inhibited ER Stress Induction by HSV-1 in SK-N-SH Cells

**Figure 5:**
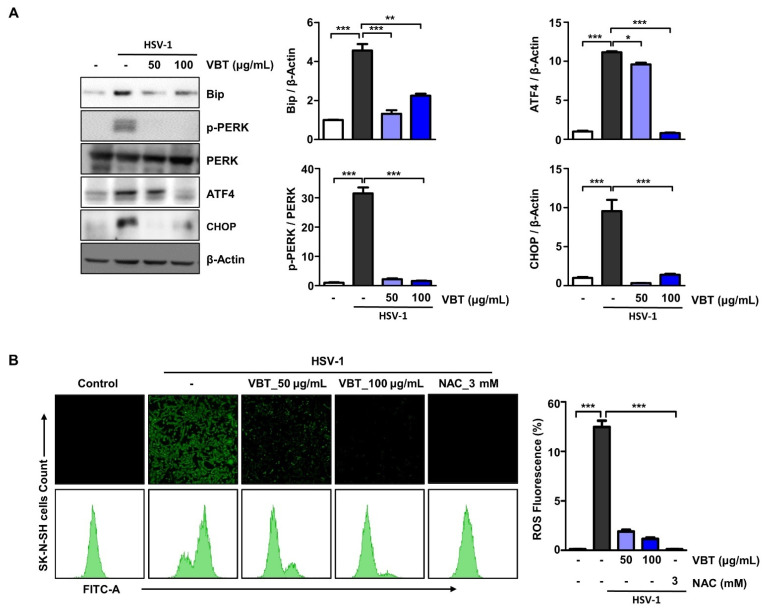
Inhibition of ER Stress and ROS by HSV-1 induction of VBT in SK-N-SH Cells. (**A**) Cells were infected with HSV-1 (MOI = 0.1) for 2 h and then replaced with fresh medium. Then, they were treated with VBT (50 μg/mL and 100 μg/mL) and ACV (10 μg/mL), and incubated for 48 h. ER stress-related factor proteins were identified by Western blotting. The expression of these proteins was normalized to that of β-Actin as a relative ratio. (**B**) Cells were infected with HSV-1 at MOI = 0.1 concentration for 2 h, then VBT (50 μg/mL and 100 μg/mL), and cultured for 24 h, followed by ROS analysis. NAC was used as a positive control. The level of ROS was measured using fluorescence microscopy and FACS. * *p* < 0.5, ** *p* < 0.01, *** *p* < 0.001 vs. HSV-1 treatment cells.

Large amounts of proteins that are produced by viral infection induce overwhelmingly folded or misfolded protein production in infected cells [21]. These changes in the protein folding environment are reported to result in the accumulation of misfolded proteins in the ER, fundamentally affecting various cellular signaling processes including energy production, inflammation, differentiation, redox homeostasis, and apoptosis [42]. To investigate the role of ER stress in the antiviral effects of VBT, Western blot analysis was used to determine the expression of BiP, PERK, ATF4, and CHOP, which are known as specific ER stress proteins. As shown in Figure 5A,B, HSV-1 increased the protein levels of BiP, PERK, ATF4, and CHOP in cells, but was inhibited in a dose-dependent manner upon VBT treatment. Next, we investigated whether VBT inhibited ROS production that was caused by ER stress in HSV-1 host cells. SK-N-SH cells were infected with HSV-1 for 2 h, treated with 50 μg/mL or 100 μg/mL of VBT in fresh medium, and cultured for 24 h. The antioxidant N-acetyl-L-cysteine, which can scavenge endogenous ROS, was used as a positive control. ROS formation was monitored using fluorescence microscopy and FACS, using the oxidant-sensitive fluorescent probe CM-DCFDA. As shown in Figure 5B, ROS generation that was induced by HSV-1 was inhibited at the concentration of VBT of 100 μg/mL. Therefore, the reduction by VBT of ER stress and ROS generation was found to contribute to its antiviral effects.

### 3.6. VBT Protects SK-N-SH Cells through CHOP-Induced Death Receptor (DR) Regulation during HSV-1 Infection

**Figure 6:**
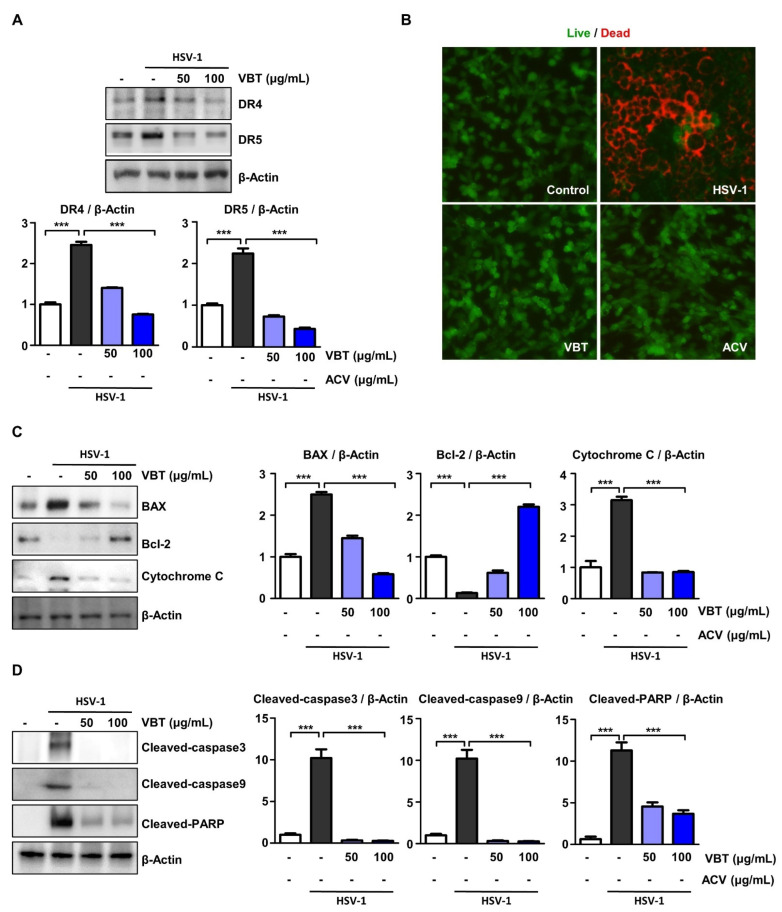
Protective effect of VBT against HSV-1 induced apoptosis in SK-N-SH cells. SK-N-SH cells were infected with HSV-1 at MOI = 0.1 for 2 h. Then, the infectious agent medium was removed and replaced with fresh medium, and VBT was added at 50 µg/mL or 100 µg/mL for 48 h. (**A**) The expression of genes that were involved in DRs makers following HSV-1 infection was measured using Western blot analysis. (**B**) Live/Dead cell staining of SK-N-SH cells. The green color represents live cells and the red color represents dead cells. Images were measured at 200× ratio using a fluorescence microscope. (**C**,**D**) The expression of genes that are involved in apoptosis makers following HSV-1 infection was measured using Western blot analysis. The expression of these proteins was normalized to that of β-Actin as a relative ratio. *** *p* < 0.001 vs. HSV-1 treatment cells.

Several studies have reported that ER stress-induced CHOP expression mediates DR activity [43,44]. Therefore, in this study, we investigated whether VBT regulates DR expression during HSV-1 infection. After cells were infected with HSV-1 for 2 h, they were treated with VBT at the indicated concentrations for 48 h, and then the expression of DR was measured. VBT was found to inhibit the expression of DR4 and DR5 in host cells that were induced by HSV-1 infection (Figure 6A). Previous studies have shown that if ER stress is irreversible, PERK-CHOP function may persist, resulting in elevated DR5 [45]. DR5 induces apoptosis by acting on the mitochondrial membrane along with BAK and BAX, which trigger the release of cytochrome C [46]. We imaged cell death using the live/dead method. As a result, CPE following HSV-1 infection was generally observed in the form of almost agglomerated cells immediately after infection, and red, that is, dead cells were observed in the infected cells (Figure 6B). We then investigated whether VBT regulates the expression of proteins that are involved in neuronal cell death upon HSV-1 infection. In SK-N-SH cells, the expression of the anti-apoptotic protein Bcl-2 following HSV-1 infection was suppressed by VBT (Figure 6C). The results that are presented in Figure 5C show that the levels of the pro-apoptotic proteins BAX and cytochrome C in HSV-1 infection were decreased by VBT. These results suggest that VBT protects brain cells from HSV-1 infection by regulating the expression of apoptotic proteins. ER stress-mediated apoptotic signaling is activated by proteolytic cleavage of caspase 3. In addition, it has been reported that caspase 3 plays an important role in the apoptosis of neurons and astrocytes in primary cells that are infected with HSV-1 [47]. As shown in Figure 6D, caspase 3 and caspase 9 were activated at 48 h, but VBT abolished their activation. We investigated the proteolytic cleavage of PARP by caspases, another hallmark of apoptosis [48]. Western blot analysis of HSV-1 infected lysates revealed PARP cleavage at 48 h, but PARP cleavage was inhibited by VBT treatment. This is associated with the activation of caspase 3 and caspase 9. Taken together, our results indicated that VBT inhibits apoptosis through the sustained activation of the UPR pathway, by inhibiting host cell formation after HSV-1 infection by its antiviral effect in neurons.

### 3.7. UHPLC-UV-HRMS Analysis of VBT

**Figure 7:**
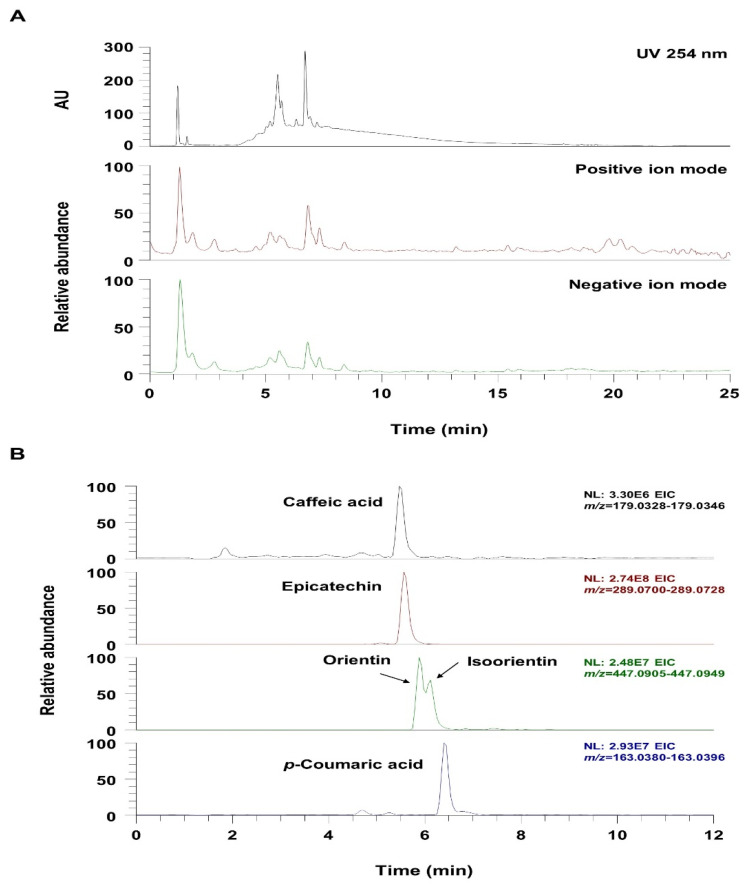
UHPLC UV HRMS analysis of *V. bracteatum*. (**A**) UV chromatogram and total ion chromatogram (TIC) of *V. bracteatum*, (**B**) Extracted ion chromatogram (EIC) of the identified phytochemicals in *V. bracteatum* in the negative ion mode.

UHPLC-UV-HRMS analysis was used to identify the phytochemicals in VBT. As shown in Figure 6 and Table 1, it was confirmed that the five phenolic components (flavonoids and phenolic acids) that were identified in VBT were consistent with the results of previous reports [49,50,51]. Figure 7A shows the UV chromatogram at wavelength 254 nm and the total ion chromatogram in positive and negative ion modes. The extracted ion chromatogram (EIC), which is a chromatogram for the specific *m*/*z* values of each analyte component, is shown in Figure 6B. All of the identified components in VBT—caffeic acid, epicatechin, orientin, isoorientin, and p-coumaric acid—were determined by comparing the retentiontime, precursor ion, and MS/MS fragment with those of the reference standards, and all components were detected in the negative ion mode (Table 1).

## 4. Discussion

HSV-1 infects the limbic structures of the central nervous system and appears to be an environmental risk factor for HSE and AD [5,6]. Acyclovir is widely used as a treatment for HSV-1 infection, but its therapeutic effect on the brain diseases that are caused by HSV-1 is limited [52]. Therefore, there is an urgent need to discover new antiviral targets and develop new drugs with antiviral mechanisms that are different from those of ACV. Natural products are important antiviral drug resources, and several extracts and pure compounds that have been isolated from plants have been shown to have anti-HSV-1 activity. Natural extracts containing phenols, flavonoids, alkaloids, saponins, steroids, glycosides, and tannins have been found to interfere with the replication of HSV-1 [53,54,55,56,57]. We investigated the antiviral and neuroprotective effects of VBT, a natural product, against HSV-1. First, we investigated the antiviral effect of VBT on HSV-1 infection in Vero and SK-N-SH cells. We then explored the mechanism by which VBT could inhibit apoptosis that is caused by HSV-1 infection in brain cells.

The HSV genome consists of three major groups, designated IE, early (E), and late (L) genes. Viruses can produce lifelong latent infection in neurons, and a virus often remains dormant until restimulated [58]. Therefore, the suppression of these genes is important to produce antiviral effects [59]. In our study, we confirmed that VBT repressed the VP16 and IE genes in both Vero and SK-N-SH cells. Using TEM photographs, we found that this repression inhibited virus production in HSV-1 infection.

We found that VBT inhibited the CPE formation that is caused by HSV-1 infection, thus protecting neuronal cells, and investigated the underlying mechanism. ER stress is one of the major stress pathways that is used by viruses to regulate replication within host cells [60,61]. Persistent ER stress that is caused by infection with HSV-1 results in higher levels of ROS under oxidative stress, due to an unbalanced redox state [62]. In particular, it has been reported that it induces the activation of PERK in human and mouse cells that are infected with the DNA virus HSV-1 [22,23] and exhibits eIF2α/ATF4 activity at the final stage of replication [24]. This activity indicates that virion assembly and escape is complete [24]. Although there have been few studies into the activity of CHOP following HSV-1 infection, it is known that the activation of ATF4 leads to the activation of CHOP [25]. We found that VBT inhibited the activity of PERK and ATF4, ER stressors induced by HSV-1, and also suppressed the expression of CHOP. Viral infections are often associated with an imbalance in the intracellular redox state of host cells, shifting them toward pro-oxidant conditions [63]. Redox alterations are useful for the virus, since many pathways that are involved in the regulation of viral replication and host responses are highly responsive to even transient changes in the redox state of the cytoplasmic environment. ROS production was also found to be inhibited.

**Figure 8:**
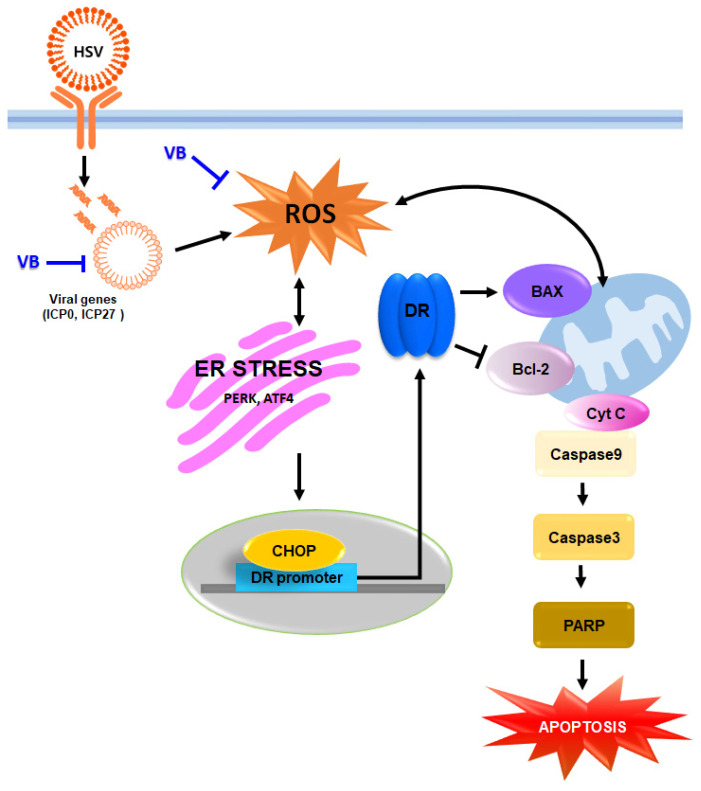
We propose an antiviral mechanism of VBT by HSV-1 infection in SK-N-SH cells. VBT exhibits an antiviral effect through the inhibition of IE expression by HSV-1 infection. Furthermore, VBT, which exhibits antiviral effects, protects SK-N-SH cells through the regulation of ROS-ER stress-mitochondrial apoptosis mechanism.

Viruses evade apoptosis for replication and production, but as a result, apoptosis is triggered in infected cells by mediators of the host immune response [12,13,14]. Previous studies have shown that infection with wild-type HSV-1 activates caspase 3 [13], the death factor DNA fragmentation factor 45 (DFF45) is completely cleaved [13], and phosphatidylserine is flipped from the inner to the outer membrane leaflet [64]. As such, most viral infections cause apoptosis or programmed cell death in infected cells, a cellular response that often occurs as a forced or unavoidable by-product of an important viral replication function. Replicable HSV-1 induces an apoptosis program, causing lysed hsv-1 to re-infect cells and display apoptotic status. [65]. In our study, VBT inhibited the proapoptotic markers BAX and cytochrome C following HSV-1 infection, and reactivated the antiapoptotic marker BCL-2. Therefore, it was confirmed that VBT inhibited the activity of caspase-3, caspase-9, and PARP (Figure 8).

## 5. Conclusions

Since HSV-1 is closely related to neuronal disease, it is important to study the antiviral effect and mechanism of VBT. We found that VBT protected SK-N-SH cells by suppressing the IE gene after HSV-1 infection and investigated the underlying mechanism. ER stress is one of the major stress pathways that viruses use to regulate replication within host cells and can activate DRs to trigger apoptosis. Based on that point, experimental results suggested that VBT could be an effective drug to protect neurons from HSV-1 infection by inhibiting ER stress induction and neuronal apoptosis after HSV-1 infection. Overall, our experimental results suggest that VBT modulates the HSV-1 signaling cascade and provides a sufficient scientific basis for investigating the therapeutic efficacy against HSV-1 infection.

## Figures and Tables

**Table 1:** Identification of phytochemicals in VBT via UHPLC-UV-HRMS.

No	tR (min)	Molecular Formula	Precursor Ion (*m*/*z*)			Error (ppm)	MS/MS Fragments (*m*/*z*)	Identifications
			Adduct	Expected	Measured			
1	5.47	C_9_H_8_O_4_	M-H	179.0350	179.0337	−0.83	135	Caffeic acid [39]
2	5.56	C_15_H_14_O_6_	M-H	289.0718	289.0714	−1.31	245, 179	Epicatechin [39]
3	5.88	C_21_H_20_O_11_	M-H	447.0933	447.0927	1.17	357, 327	Orientin [40,41]
4	6.11	C_21_H_20_O_11_	M-H	447.0933	447.0928	1.13	357, 327	Isoorientin [40,41]
5	6.39	C_9_H_8_O_3_	M-H	163.0401	163.0388	−1.21	119	p-Coumaric acid [39]

Compared retention time, precursor ion, and MS/MS fragments to reference standards.

## Data Availability

All the data is available within the article and Supplementary Materials.

## Supplementary Materials

The following supporting information can be downloaded at: https://www.mdpi.com/article/10.3390/antiox11091773/s1, Figure S1: Full scan mass spectrum and MS2 spectrum of caffeic acid; Figure S2: Full scan mass spectrum and MS2 spectrum of epicatechin; Figure S3: Full scan mass spectrum and MS2 spectrum of orientin; Figure S4: Full scan mass spectrum and MS2 spectrum of isoorientin; Figure S5. Full scan mass spectrum and MS2 spectrum of *p*-coumaric acid.
