# Supplementary material for: Vaccinium bracteatum Thunb Extract Inhibits HSV-1 Infection by Regulating ER Stress and Apoptosis
Source: Antioxidants (Basel). 2022 Sep 8;11(9):1773. doi: 10.3390/antiox11091773 (PMC9495922; doi:10.3390/antiox11091773)
Supplement: Supplementary file 1 [file antioxidants-11-01773-s001.zip › antioxidants-1848073-supplementary.pdf]

Supplementary materials

Q-Exactive quadrupole-orbitrap mass spectrometer condition

MS/MS analysis was conducted using a Q-Exactive quadrupole-orbitrap mass spectrometer that equipped with a heated electrospray ionization (HESI) interface. The ionization source was optimized using the following parameters: positive and negative ion-switching mode; spray voltage, 3.5 kV; capillary temperature, 350°C; sheath gas pressure, 40 arbitrary units (au); auxiliary gas pressure, 10 au; and S-lens RF level 50. MS spectra were acquired in full MS and dd-MS^2 (top N) scan mode. This mode acquired full MS scans followed by a set of data-dependent (dd) scans with fragmentation energy applied. The full MS acquisition parameters were set as follows: ion scan range 100-1500 m/z; resolution of MS scan, 70000; automatic gain control (AGC) target, 1.0E6; maximum injection time (IT), 100 ms; and profile spectrum data type. The MS2 optimized acquisition parameters were as follows: resolution of MS/MS scan, 17500; AGC target, 1.0E5; maximum IT, 50 ms; count of loop, 10; count of MSX, 1; and normalized collision energy, 25 eV. All data acquisition and analysis were performed using Xcalibur v.4.2 and Tracefinder v.4.0 software (Thermo Fisher Scientific).

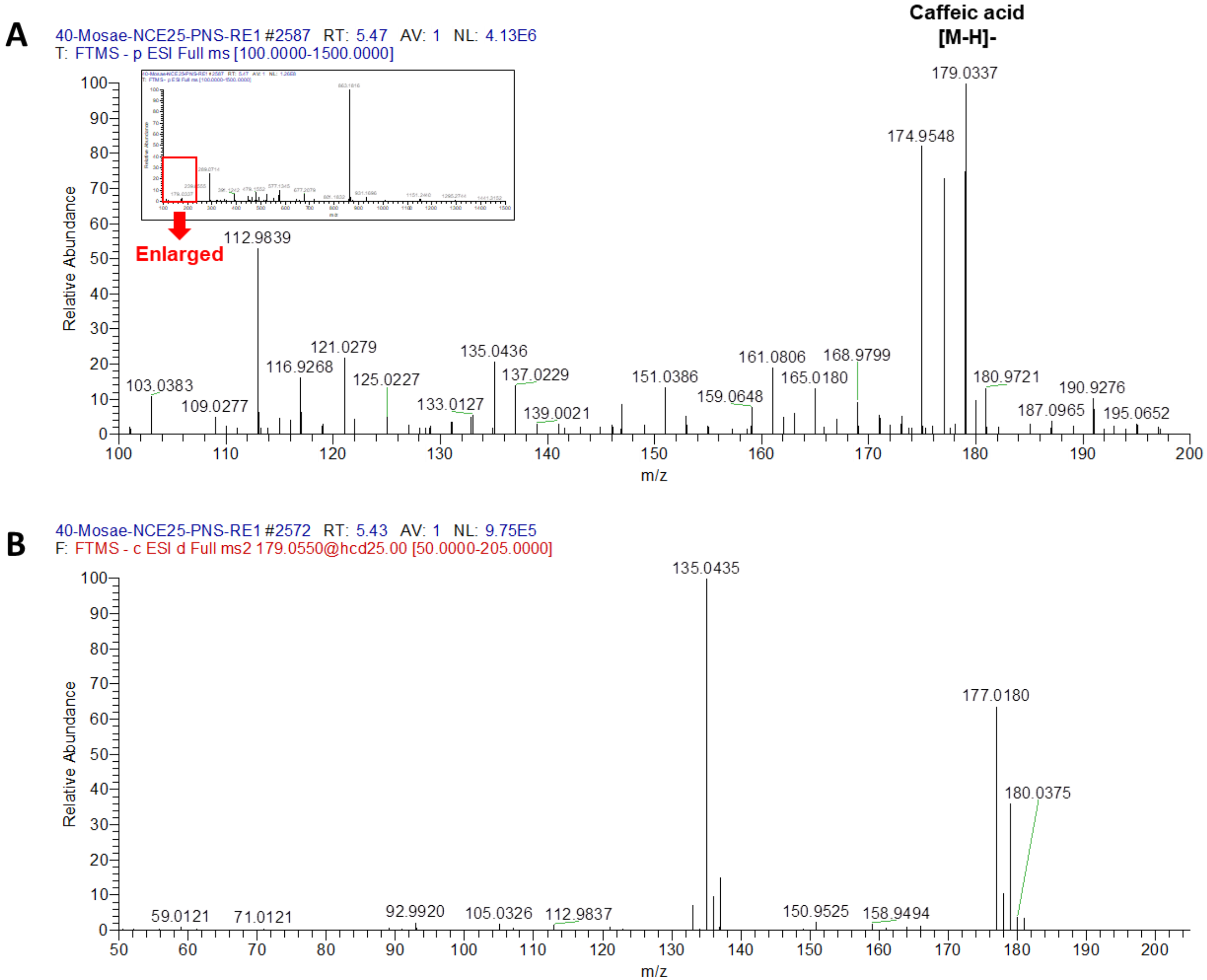

Figure S1. Full scan mass spectrum (A) and MS2 spectrum (B) of caffeic acid

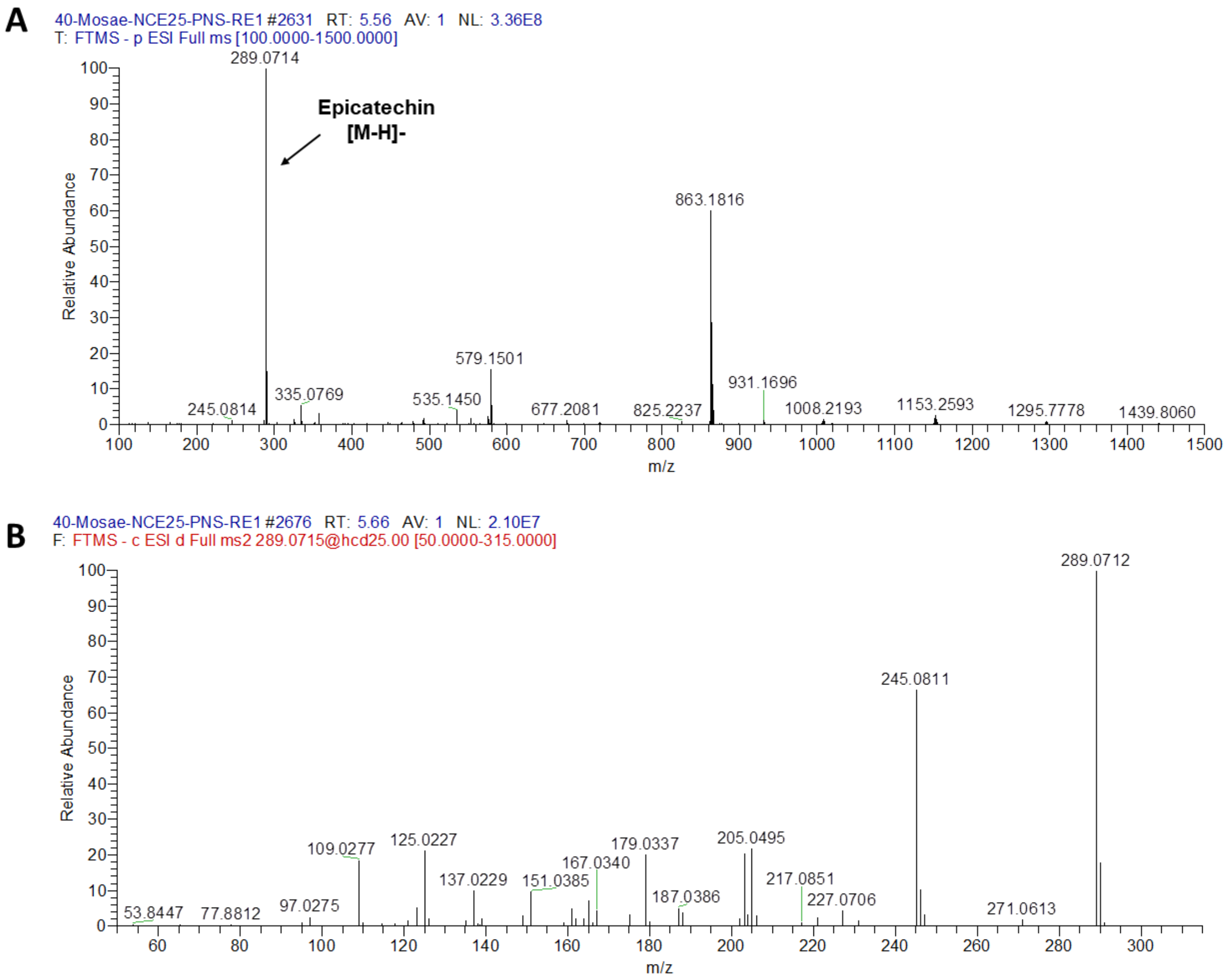

Figure S2. Full scan mass spectrum (A) and MS2 spectrum (B) of epicatechin

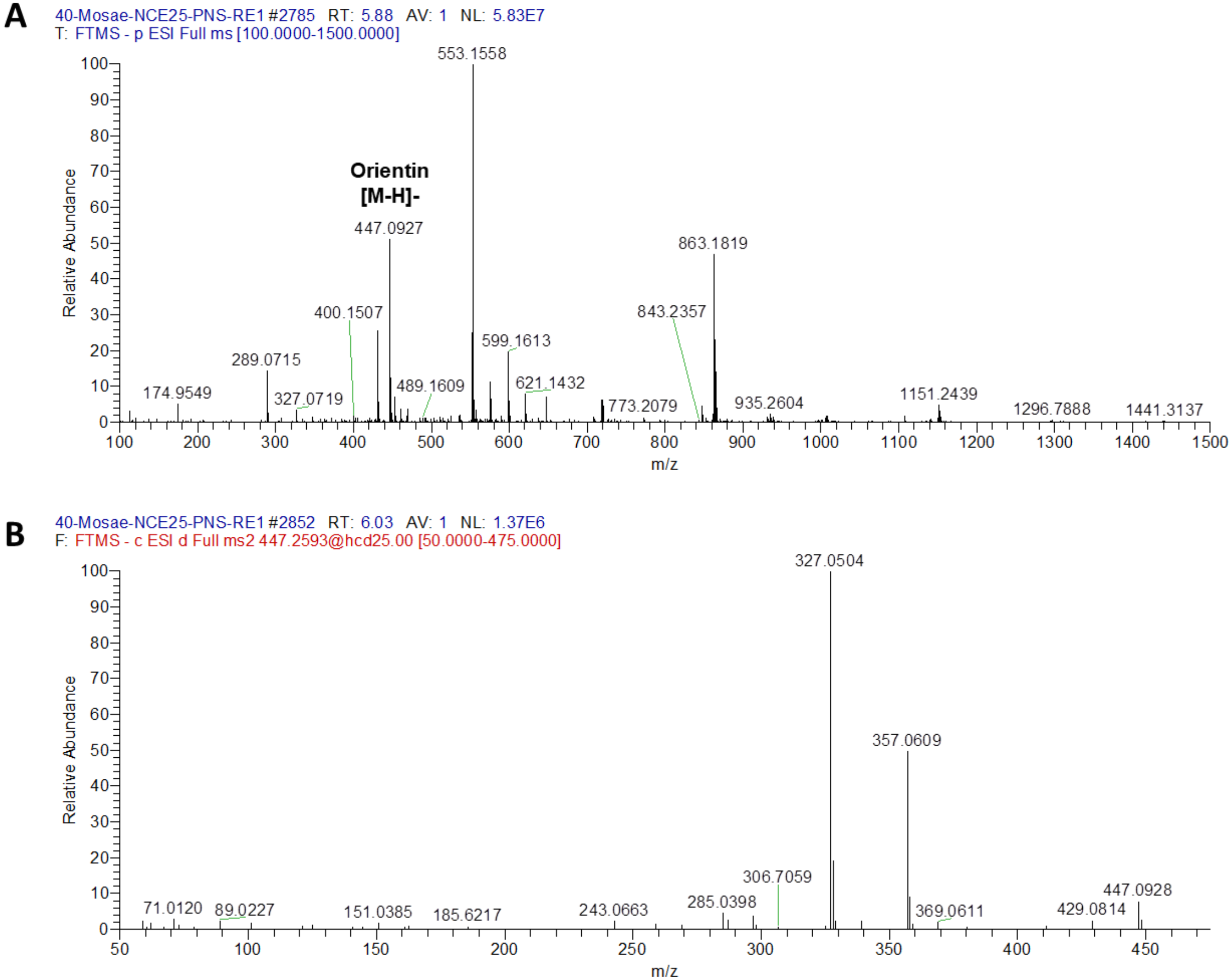

Figure S3. Full scan mass spectrum (A) and MS2 spectrum (B) of orientin

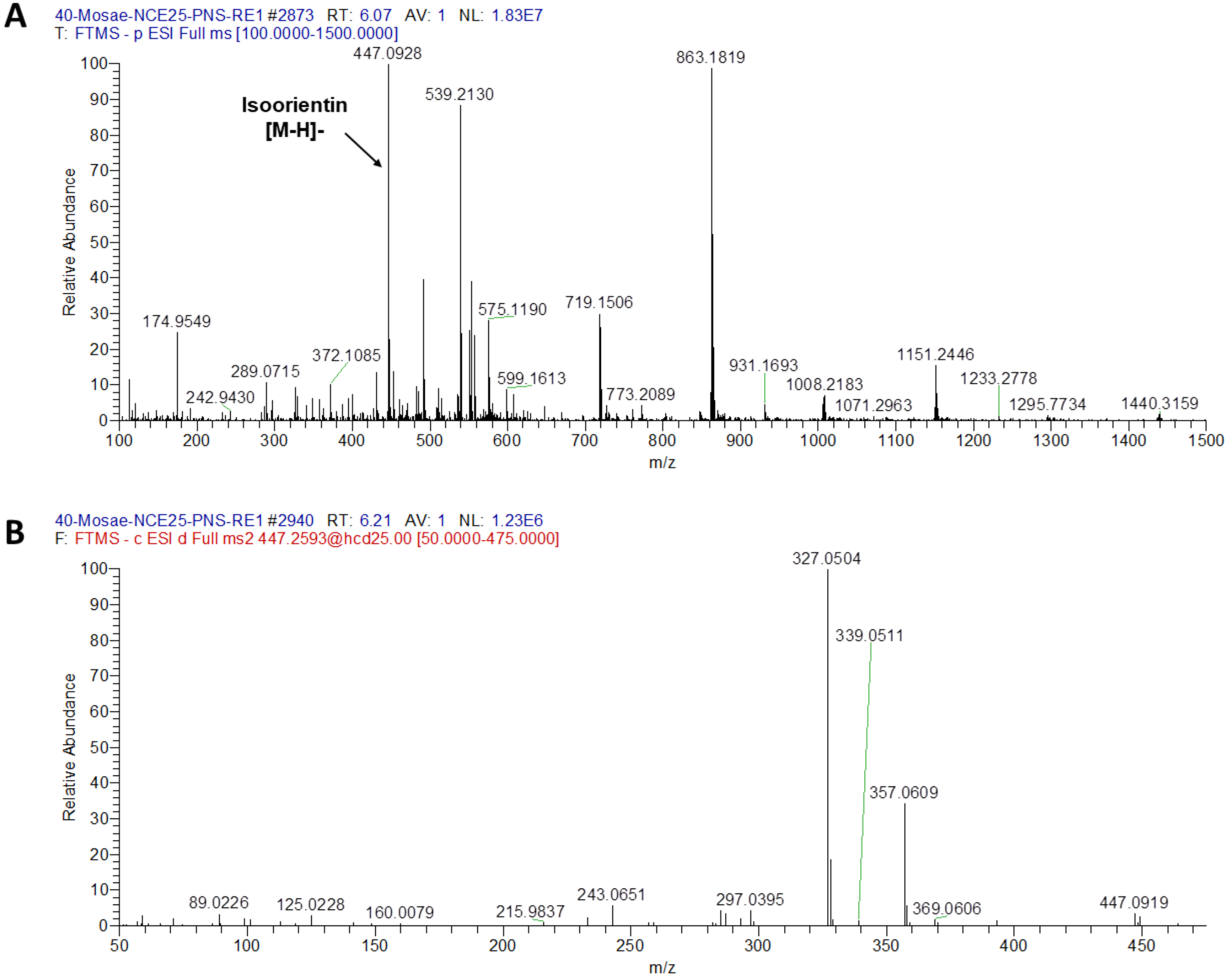

Figure S4. Full scan mass spectrum (A) and MS2 spectrum (B) of isoorientin

A

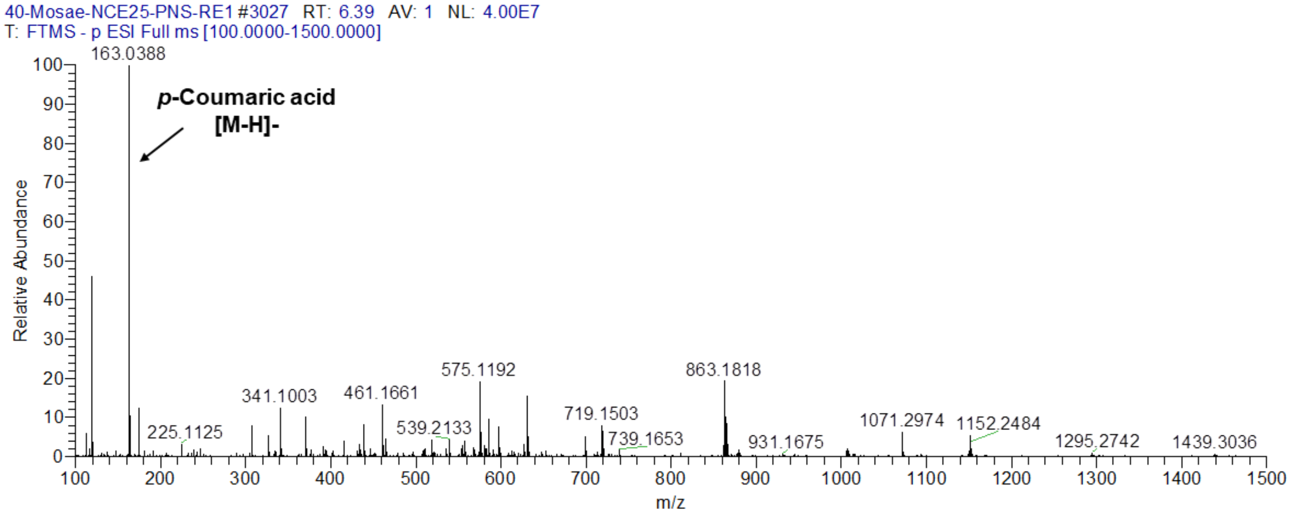

B

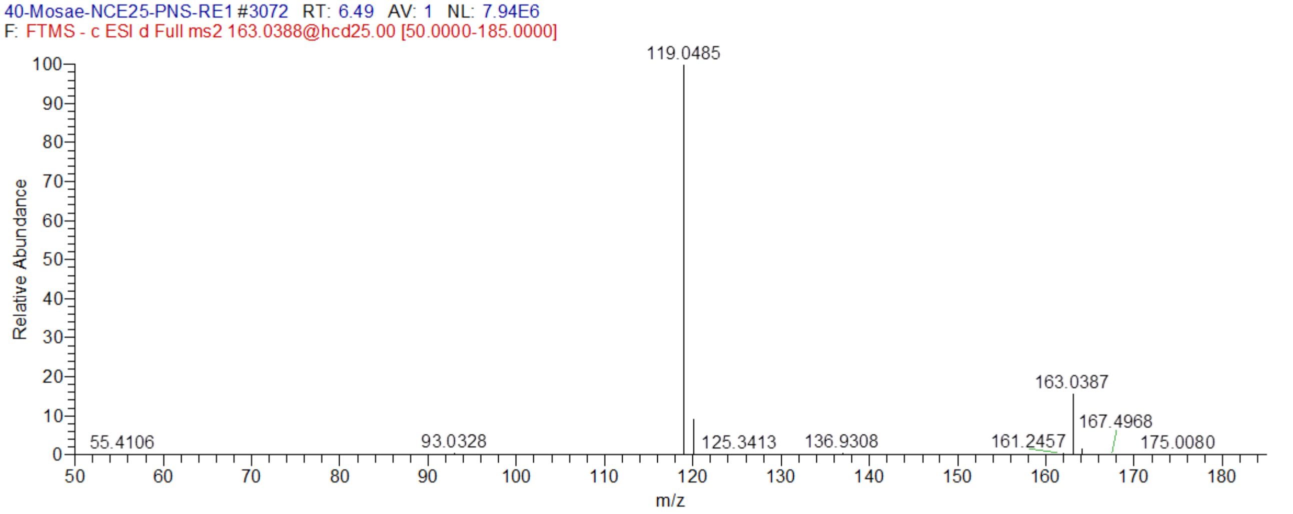

Figure S5. Full scan mass spectrum (A) and MS2 spectrum (B) of *p*-coumaric acid
